# Thinking outside the box: leveraging endoscopic ultrasound–guided portal pressure gradient for comprehensive risk stratification in liver transplantation candidates

**DOI:** 10.1016/j.igie.2026.02.008

**Published:** 2026-02-20

**Authors:** Abdulmalik Saleem, Ammad Chaudhary, Kevin Harris, Yara Dababneh, Yervant Ichkhanian, Reena Salgia, Andrew Watson

**Affiliations:** 1 Department of Internal Medicine, Henry Ford Hospital, Detroit, Michigan, USA; 2 Department of Gastroenterology and Hepatology, Mayo Clinic, Rochester, Minnesota, USA; 3 Department of Gastroenterology and Hepatology, Henry Ford Hospital, Detroit, Michigan, USA; 4 Department of Gastroenterology, Indiana University School of Medicine, Indianapolis, Indiana, USA

## Abstract

**Background and Aims:**

Coronary artery disease (CAD) is common in liver transplantation (LT) candidates and may require coronary artery bypass grafting (CABG). Risk stratification is challenging in cirrhosis because histology alone does not reliably predict clinically significant portal hypertension (CSPH). Endoscopic ultrasound–guided portal pressure gradient (EUS-PPG) provides minimally invasive, real-time hemodynamic assessment. The aim of this study was to evaluate the role of EUS-PPG in perioperative risk stratification among LT candidates requiring CABG.

**Methods:**

We retrospectively reviewed 3 LT candidates with hepatocellular carcinoma (HCC) as the indication for transplant (2021-2023). All had obstructive multivessel CAD not amenable to percutaneous coronary intervention and were evaluated for CABG using EUS-PPG when feasible.

**Results:**

Two patients with biopsy-proven cirrhosis had PPG <6 mm Hg, excluding CSPH; both underwent CABG and successful LT. In 1 patient, PPG could not be obtained because of anatomical distortion; CABG proceeded urgently, but the patient died postoperatively from stroke and aspiration pneumonia, which were unrelated to portal hypertension.

**Conclusions:**

EUS-PPG may be safe and clinically informative, complementing the Model for End-Stage Liver Disease–based risk stratification and histology to refine perioperative risk assessment in LT candidates with HCC and CAD, although additional cases and further study are needed.

## Background

The rapid advancement of endoscopic techniques has proven fertile ground for innovation, particularly in the complex evaluation of liver transplantation (LT) candidates. The presence of chronic liver disease with cirrhosis is associated with a higher comorbidity index than the noncirrhotic population.^1^^,^^2^ It is reported that nearly 25% of LT candidates have asymptomatic obstructive coronary artery disease (CAD).^3^ Multivessel CAD can increase the risk of mortality and morbidity after LT.^4^ Thus, obstructive coronary CAD can affect LT eligibility and, in some cases, be prohibitive unless a patient is successfully revascularized.^5^^,^^6^ Cardiothoracic surgery before LT is often challenged by the increased risk of postoperative morbidity and mortality from decompensated cirrhosis.^7^^,^^8^ In these settings, accurate risk assessment is critical to stratify patients' suitability for preoperative coronary artery bypass grafting (CABG). Biochemical, imaging, and elastography assessments may have mixed results for portal hypertension (PH) assessment in compensated cirrhosis and can affect risk stratification for clinically significant PH (CSPH).

Portal pressure gradient (PPG) reflects the severity of PH and is a robust prognostic factor in chronic liver disease, complementary to Model for End-Stage Liver Disease (MELD)-based risk stratification.^1^^,^^4^, ^5^, ^6^ Endoscopic ultrasound–guided PPG (EUS-PPG) offers a minimally invasive, real-time method to directly measure portal pressures and, within the same session, obtain elastography, screen for varices, and perform endoscopic liver biopsy, enhancing PH characterization and clinical decision-making.^9^ Here, we report 3 LT candidates with severe CAD in whom EUS-PPG informed CABG decision-making and subsequent transplant planning (Table 1).

**Table 1:** EUS-PPG and cardiac evaluation in patients with cirrhosis undergoing liver transplantation evaluation

Patient	Etiology of cirrhosis	MELD 3.0	Degree of fibrosis	Committee recommendation before EUS-PPG	Mean PPG (mm Hg)	Cardiac intervention	Committee recommendation after EUS-PPG	Outcome
1	HCV cirrhosis with HCC	8	Frequent bridging fibrosis with nodular formation	Cardiac intervention, followed by transplant committee re-evaluation	5.3	3-vessel CABG	Listed for LT	Successful OLT
2	Hemochromatosis/ETOH with HCC	8	Scattered bridging fibrosis with several nodular formations	Cardiac intervention, followed by transplant committee re-evaluation	4.0	3-vessel CABG	Listed for LT	Successful OLT
3	ETOH cirrhosis with HCC	8	Portal and periportal fibrosis with focal bridging	Cardiac intervention, followed by transplant committee re-evaluation	Not performed given the diminutive hepatic vein and altered anatomy/distortion from mass	3-vessel CABG	Evaluation ended as the patient died 57 days after CABG	Deceased

*CABG*, Coronary artery bypass grafting; *ETOH*, alcohol; *EUS-PPG*, endoscopic ultrasound–guided portal pressure gradient; *HCC*, hepatocellular carcinoma; *HCV*, hepatitis C virus; *LT*, liver transplantation; *MELD*, Model for End-Stage Liver Disease; *PPG*, portal pressure gradient; *OLT*, orthotopic liver transplantation.

## Methods

This retrospective series (2021-2023) included LT candidates at a quaternary referral center who had obstructive multivessel CAD for which percutaneous coronary intervention was unsuitable and were being evaluated for CABG before LT. Institutional Review Board approval was obtained from Henry Ford Health. Demographic, clinical, histologic, and procedural data were abstracted from the medical record.

EUS-PPG was initiated with the insertion of a linear echoendoscope positioned in the stomach or duodenum. Under Doppler guidance, a 25-gauge Cook EchoTip Insight fine-needle aspiration needle (Cook Medical, Bloomington, Ind, USA) was advanced sequentially into a hepatic vein and then the portal vein, with the needle connected via noncompressible tubing to a calibrated pressure transducer; pressures were recorded, and the PPG was calculated as portal minus hepatic venous pressure. All procedures were performed with patients under general anesthesia. Portal and hepatic venous pressures were measured in triplicate and averaged. The technique is reproducible and is not technically complex.^9^

## Results

### Case 1

A 72-year-old man with hepatitis C virus–related cirrhosis and hepatocellular carcinoma (HCC) (MELD 3.0 = 8) underwent EUS-PPG as part of LT evaluation, with HCC as the indication for listing (Table 1). Imaging revealed 2 lesions measuring 1.3 cm in segment 7 and 1.5 cm at the segment 7/8 junction, both previously treated with transarterial radioembolization, which were not evaluated during endoscopic ultrasound (EUS). Concurrent biopsy of nonlesional liver parenchyma during portal pressure measurement demonstrated frequent bridging fibrosis with nodular formation. The PPG measured 5.3 mm Hg. Given the presence of obstructive CAD not amenable to percutaneous coronary intervention, the transplant committee recommended CABG, which was successfully completed, after which the patient was listed and ultimately underwent orthotopic LT without adverse events.

### Case 2

A 65-year-old man with hemochromatosis, alcohol (ETOH)-associated cirrhosis, and HCC (MELD 3.0 = 8) underwent EUS-PPG as part of LT evaluation for HCC (Table 1). Cross-sectional imaging identified a 3.0-cm lesion in segment 7/9 and a 1.5-cm lesion in the inferior lobe, both previously treated with percutaneous microwave ablation. To note, these lesions were not assessed by EUS. During portal pressure measurement, EUS-guided biopsy of nonlesional liver parenchyma demonstrated scattered bridging fibrosis with several nodular formations. The PPG measured 4.0 mm Hg. Given these findings, the patient underwent successful CABG and subsequently underwent successful LT.

### Case 3

A 69-year-old man with ETOH-associated cirrhosis and HCC (MELD 3.0 = 8) presented for LT evaluation for HCC. The patient had a 2.4-cm lesion and a 1.3-cm lesion, both located in segment 2 (Table 1). EUS-PPG was attempted; however, because of diminutive hepatic vein caliber, measurement could not be obtained and was thus not pursued with hepatic portal venous gradient. Given histologic findings without cirrhosis (Ishak stage 3 fibrosis) and no endoscopic evidence of portal hypertension, CABG was performed without EUS-PPG measurement. The patient subsequently died 57 days after CABG from postoperative aspiration pneumonia and subsequent acute respiratory distress syndrome.

## Discussion

PPG values between 6 and 9 mm Hg are generally considered mild PH, whereas ≥10 mm Hg defines CSPH.^9^ In cases 1 to 2, despite histologic cirrhosis, PPG was <6 mm Hg, indicating compensated cirrhosis without CSPH and supporting the decision to proceed with CABG before LT. These findings reinforce that histologic cirrhosis does not uniformly imply CSPH and that direct pressure measurement can refine perioperative risk assessment in LT candidates with CAD.^5^^,^^6^^,^^9^

All 3 patients had low MELD 3.0 scores, emphasizing that LT candidacy was oncologic rather than driven by decompensation. MELD may under-represent perioperative risk in compensated patients, whereas PPG adds complementary prognostic insight.^4^, ^5^, ^6^^,^^8^ The 2 patients with normal PPG proceeded with CABG because their PPG was normal. EUS-PPG did not change the decision to pursue CABG but refined perioperative risk stratification by confirming the absence of CSPH in compensated cirrhosis. The third case highlights a real-world limitation of EUS-PPG when unfavorable hepatic anatomy precludes measurement.

EUS-PPG was selected over a transjugular interventional radiology approach because it enables minimally invasive, real-time portal pressure assessment during a single endoscopic session without the need for separate vascular access or fluoroscopy, which is particularly advantageous in LT candidates already undergoing EUS-based evaluation.^9^ EUS-PPG was performed during a single EUS session, meaning concurrent with liver parenchymal assessment with biopsy when indicated. Future work should validate these findings prospectively and evaluate integration with multimodal assessment tools such as magnetic resonance imaging, elastography, and biomarkers, including fibrosis-4 index and Mac-2 binding protein glycosylation isomer.

## Patient Consent Statement

Consent was not required for this study. Patient identifiers were not used in compliance with the Health Insurance Portability and Accountability Act.

## Disclosure

The following authors disclosed financial relationships: R. Salgia: Speaker for Cook Medical. A. Watson: Speaker for Cook Medical. All other authors disclosed no financial relationships.
